# Non-Hodgkin Lymphoma of the External Auditory Canal: A Rare Primary Involvement

**DOI:** 10.7759/cureus.75040

**Published:** 2024-12-03

**Authors:** Sandra R Sousa, Cátia M. L Pereira, Patrícia Ferraz, Miriam Blanco

**Affiliations:** 1 Internal Medicine Department, Unidade Local de Saúde do Nordeste, Bragança, PRT; 2 Hematology Department, Unidade Local de Saúde de Trás-os-Montes e Alto Douro, Vila Real, PRT

**Keywords:** acute otitis media, external ear, facial palsy, non-hodgkin lymphomas, primary lymphoma

## Abstract

The authors describe a rare case of non-Hodgkin lymphoma (NHL) with primary involvement of the external auditory canal (EAC) and subsequent dissemination to the central nervous system, initially manifesting as a benign ear infection. This case highlights the importance of considering differential diagnoses in patients with persistent or worsening symptoms unresponsive to empirical treatment. A 53-year-old man presented with a one-week history of aural fullness, otalgia, and otorrhea in the left ear. The first examination of the EAC showed no masses or lesions, but the tympanic membrane was bulging and erythematous. A diagnosis of acute otitis media was established, and the patient started empirical antibiotic treatment. However, the patient’s symptoms worsened during the course of therapy. A brain MRI revealed a lesion occupying the left EAC, extending to the retro-auricular region, surrounding the mastoid. The patient underwent an excisional biopsy of a postauricular lesion, and histological examination was compatible with diffuse large B-cell lymphoma. Chemotherapy was initiated, but at the end of the treatment, the disease progressed. The patient subsequently underwent second-line chemotherapy and was referred for a transplant consultation. In this case, the NHL of the EAC initially presented as an apparently benign ear infection, masking the underlying severity of the disease. In summary, NHL presents a significant challenge, not only because of their heterogeneous and non-specific clinical presentation which can delay diagnosis but also because of the difficulty in treatment. This report emphasizes the need for high clinical suspicion and a comprehensive diagnostic approach to improve the prognosis of patients with NHL in atypical locations.

## Introduction

Non-Hodgkin lymphomas (NHLs) are a heterogeneous group of lymphoproliferative malignancies, divided into B-cell lymphomas and T-cell or natural killer lymphomas [1]. NHL commonly manifests as persistent painless lymphadenopathy, sometimes accompanied by systemic symptoms like night sweats, prolonged fever, unintentional weight loss, and symptoms related to the affected organ [1].

Approximately 25% of NHLs present with a primary extranodal location [2], and among these cases, around 15-25% involve the head and neck region, namely, the nasopharynx, larynx, and lacrimal glands [2,3]. Primary NHLs of the auditory canal are rare, and when they do occur, they typically involve the middle ear [4]. The external auditory canal (EAC) involvement is even rarer and has been only sparsely reported in the literature [3,4].

The initial presentation of primary NHL of the ear canal is non-specific and often resembles acute otitis media (AOM) or external otitis, most commonly manifesting as hearing loss, a sensation of aural fullness, otalgia, otorrhea, or otorrhagia [3,4]. When there is no response to empirical antibiotic treatment and worsening of the initial clinical presentation, it is essential to reconsider the diagnosis and order imaging studies [3]. Computed tomography (CT) and magnetic resonance imaging (MRI) are crucial for diagnosis, although histological examination is required to confirm NHL [2,4].

With this article, the authors aim to report a rare case of diffuse large B-cell lymphoma (DLBCL) with primary infiltration of the EAC with secondary involvement of the central nervous system (CNS), which initially presented as a benign ear infection.

## Case presentation

**Figure 1:**
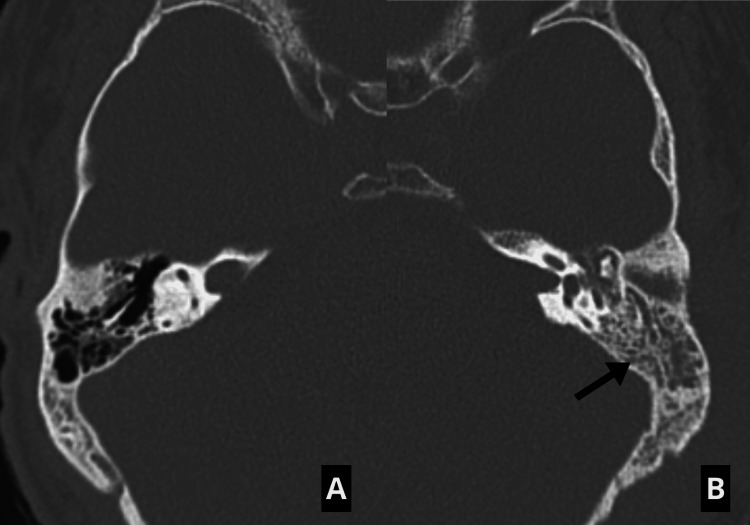
Computed tomography scan of the temporal bone (axial view) A: The right middle ear cavity and mastoid are normally aerated, with intact ossicles. B: The left side shows inflammatory material dispersed throughout the mastoid and much of the tympanic cavity (arrow), along with enlargement and mild erosion at the superior limit of the external auditory canal.

A 53-year-old independent man with a history of hyperuricemia and atrial fibrillation, treated with apixaban and allopurinol, presented with a one-week history of aural fullness, otalgia, and otorrhea in the left ear. On physical examination, the patient was hemodynamically stable and afebrile, with a normal neurological examination. Examination of the EAC did not reveal any masses or lesions, but the tympanic membrane was bulging and erythematous. An initial diagnosis of AOM was made, and the patient started empirical antibiotic therapy with amoxicillin 875 mg and clavulanic acid 125 mg orally, every 12 hours. On the fifth day of treatment, the patient's symptoms worsened, accompanied by left-sided peripheral facial palsy (PFP), horizontal nystagmus to the left, vertigo, vomiting, and fever. Due to the suspicion of complications such as malignant otitis externa, otomastoiditis, or potentially labyrinthine fistula, a CT scan of the temporal bone was performed. It revealed inflammatory material dispersed throughout the left mastoid and much of the tympanic cavity (Fig. 1), along with an extensive epicranial and subcutaneous temporo-occipital and upper cervical component, including the EAC, which was enlarged with apparent slight erosion at its superior limit, findings consistent with left otomastoiditis.

**Figure 2:**
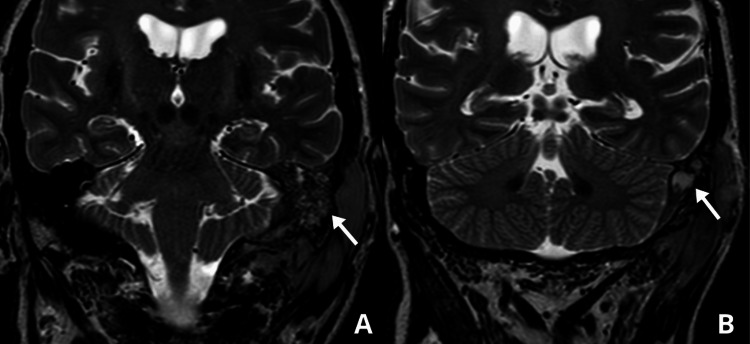
Pre-biopsy brain MRI (coronal view) Heterogeneous lesion (2 x 1.3 cm) occupying the left external auditory canal with extension to the retroauricular region, surrounding the mastoid (arrow in panel A), and involving the facial nerve at the stylomastoid foramen (arrow in panel B).

The patient was admitted to the ward and started on antibiotic therapy with ceftriaxone 2 g once daily and metronidazole 500 mg every eight hours, as well as systemic corticosteroids with dexamethasone 6 mg once daily. On the first day of admission, a brain MRI was performed and revealed a heterogeneous lesion occupying the left EAC, measuring 2 x 1.3cm, extending to the retro-auricular region, surrounding the mastoid (Fig. 2A), without signs of bone erosion but infiltrating the upper cervical fatty tissues and involving the left internal jugular vein and the left facial nerve at the stylomastoid foramen (Fig. 2B) and along its extracranial course. Given the lesion's involvement of the EAC and infiltration of adjacent structures revealed on the MRI, as well as the bone erosion observed on the CT, the hypotheses of lymphoma or malignant otitis externa, including those caused by fungal infection, were considered.

Following these diagnoses, two biopsies of the EAC lesion were performed, but both were inconclusive; therefore, an excisional biopsy of the retroauricular lesion was performed, with tissue submitted for histopathology, flow cytometry, microbiology, and mycology. Culture testing was negative and antibiotic therapy was discontinued. Histopathology confirmed a diagnosis of diffuse large B-cell lymphoma, based on the WHO-HAEM5 classification [5], with BCL-6 rearrangement, BCL-2, and MYC in their germline configuration, and negative for CD10 and MUM1. The Ki-67 nuclear proliferation index was greater than 90%, indicating a high rate of neoplastic cell proliferation. A chest and abdominopelvic contrast-enhanced CT (CECT) was performed, revealing no additional lesions. A lumbar puncture was carried out, and cerebrospinal fluid analysis showed no immunophenotypic evidence of lymphoma. HIV tests for types 1 and 2 were negative.

**Figure 3:**
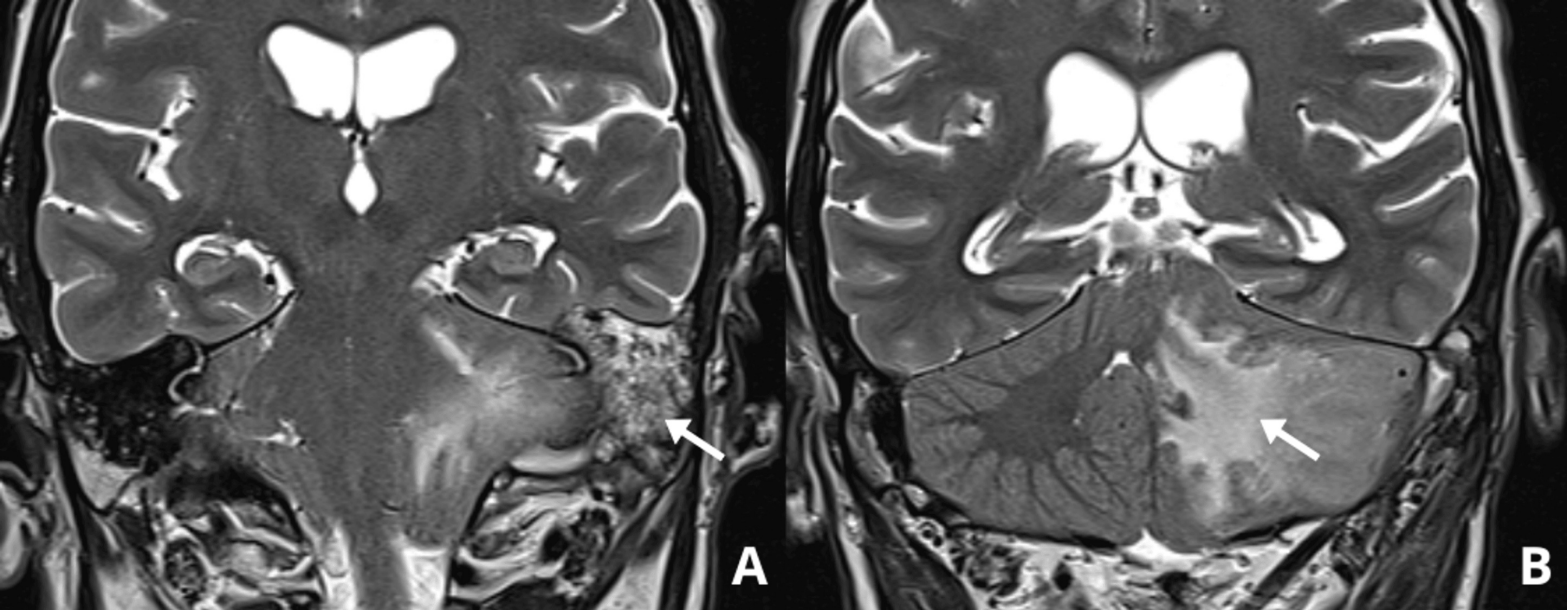
Brain MRI after six cycles of chemotherapy (coronal view) In the comparative study, progressive growth of a heterogeneous lesion (2.6 x 1.6 cm) occupying the left external auditory canal and filling the mastoid (arrow in panel A). A new expansive lesion in the left cerebellar hemisphere, measuring 3.7 cm (arrow in panel B), causing compression of the fourth ventricle.

With a diagnosis of primary DLBCL of the EAC, stage I-E, and an International Prognostic Index (IPI) score of 2, indicating low-intermediate risk, the patient was proposed and accepted to start chemotherapy, according to the R-CHOP protocol (rituximab, cyclophosphamide, doxorubicin, vincristine, and prednisolone). The patient completed six cycles of chemotherapy, with a resolution of neurological symptoms and pain complaints. One month after the last chemotherapy cycle, the patient experienced a recurrence of symptoms, including left PFP, vertigo, tinnitus, and vomiting. The comparative brain MRI study showed progressive growth of a heterogeneous lesion (2.6 x 1.6 cm) occupying the left external auditory canal and filling the mastoid (Fig. 3A), as well as a new expansive lesion in the left cerebellar hemisphere, measuring 3.7 cm (Fig. 3B), causing compression of the fourth ventricle and in close contact with the left mastoid. The patient underwent a follow-up chest and abdominopelvic CECT, with no new lesions.

After a discussion with Neurosurgery, it was decided not to repeat the biopsy due to the associated risks and the difficult-to-access location. In light of DLBCL progression with secondary CNS involvement, the patient began a new chemotherapy cycle under the MATRix protocol (high-dose methotrexate, cytarabine, thiotepa, and rituximab). The patient completed two cycles of salvage chemotherapy and underwent stem cell collection in preparation for an autologous transplant. Unfortunately, despite these efforts, the patient ultimately passed away nine months after the diagnosis.

## Discussion

NHLs are a group of lymphoproliferative malignancies that primarily affect both lymphoid and hematopoietic tissues but may also involve other organs [1]. Among the NHL of the head and neck region, the majority are localized in Waldeyer’s ring or in the lymph nodes of the neck [3]. NHLs of the ear are rarely reported and usually present as therapy-resistant otitis [4] and require high clinical suspicion to avoid delays in diagnosis and to ensure appropriate management [3]. In this case, the NHL of the EAC initially presented as an apparently benign ear infection, masking the underlying severity of the disease.

DLBCLs constitute approximately 30-40% of all NHLs and are the most common histologic subtype [6]. In addition to histologic classification, further testing for rearrangements of MYC, BCL-2, and BCL-6 is important, as these genetic alterations are associated with poor prognosis [1,6], particularly the “double-hit” MYC/BCL-2, currently classified by the WHO-HAEM5 as high-grade B-cell lymphoma [5].

The Ann Arbor staging system was developed for Hodgkin lymphoma, but it has been widely accepted for stratifying patients with NHLs. However, this system does not include important factors that predict treatment outcomes [7]. Thus, the prognosis for patients with DLBCLs is also determined by risk assessment using the IPI [8].

NHLs remain an important challenge, not only because of their heterogeneous presentation that can delay diagnosis [1,9] but also because of the difficulties in treatment [1,4]. The treatment of primary NHLs of the EAC continues to be complex not only due to the few cases reported but also to the lack of specific guidelines [2]. The standard first-line treatment for DLBCLs is chemo-immunotherapy with R-CHOP, with or without radiation, depending on the disease stage and clinical risk factors [10]. Nowadays, the main purpose of surgery is to establish a histologic diagnosis [1]. For stage I/II DLBCL, the treatment involves three cycles of CHOP-R followed by involved-field radiotherapy [11]. In patients who wish to avoid radiotherapy, the recommended treatment is 4 cycles of CHOP-R if the IPI is 0 or six cycles of CHOP-R if the IPI is 1 or 2, as was decided in our case. For advanced-stage III/IV DLBCL, which accounts for up to 70% of patients with DLBCL, the first-line treatment remains R-CHOP [11]. In high-grade B-cell lymphomas, outcomes with R-CHOP protocol are typically poor [6]. In these cases, treatment with cyclophosphamide, etoposide, vincristine, prednisone, and rituximab (CEOP-R), or etoposide, prednisone, vincristine, cyclophosphamide, doxorubicin, and rituximab (EPOCH-R), may be alternative options [11].

Although chemotherapy with the R-CHOP protocol is both safe and effective, up to 50% of patients may experience relapse, with approximately 80% of these failures occurring within the first 18 months [6,12]. In cases of lack of response and disease progression, salvage chemotherapy with more aggressive regimens, such as rituximab, ifosfamide, carboplatin, and etoposide (R-ICE), or other regimens like rituximab, dexamethasone, cytarabine, and cisplatin (R-DHAP), followed by autologous stem cell transplantation, should be considered [1,11-12].

While germinal center B-cell-type DLBCLs typically respond well to standard chemotherapy, this patient exhibited a high proliferation rate (Ki-67 >90%), which likely contributed to rapid tumor growth and aggressive behavior, even in the absence of a “'double-hit”' rearrangement (MYC/BCL-2), which is generally associated with poor prognosis [5]. Furthermore, although no clear signs of CNS involvement were detected at admission, the disease's aggressive nature and high proliferation rate suggest the possibility of early CNS dissemination, a condition where the R-CHOP regimen is notably ineffective [11]. To address both systemic lymphoma and potential CNS involvement, the MATRix protocol was initiated in our patient, as R-CHOP is not recommended for treating CNS lymphoma [11,13]. In younger patients, alternating cycles of the MATRix regimen with R-ICE may offer a more comprehensive approach [13].

The prognosis for DLBCL with secondary CNS involvement remains particularly poor, with an expected survival of less than six months [13]. Together, these factors underline the challenges faced in this case and explain the patient's unfavorable treatment outcome.

Although there has been substantial progress in understanding the genetic and molecular characteristics of DLBCL, this knowledge has yet to lead to significant improvements in frontline treatments in high-risk patients and refractory disease.

## Conclusions

While primary NHLs of the EAC remain extremely rare, this case reinforces the critical need for sustained clinical vigilance and a comprehensive diagnostic approach. This case also emphasizes the importance of considering differential diagnoses in patients presenting with persistent or worsening symptoms, unresponsive to empirical treatment. Early recognition and prompt intervention are essential for better outcomes. The literature should continue to document and analyze these cases to improve the management of these patients.
